# Crossing the Rubicon: Adipose Tissue Autophagy Breaks Out NAFLD

**DOI:** 10.1016/j.jcmgh.2021.08.006

**Published:** 2021-08-27

**Authors:** Jieun Kim, Ekihiro Seki

**Affiliations:** Karsh Division of Gastroenterology and Hepatology, Department of Medicine, Cedars-Sinai Medical Center, Los Angeles, California

Nonalcoholic fatty liver disease (NAFLD), a hepatic manifestation of the metabolic syndrome, is the leading cause of chronic liver disease. NAFLD is known to be closely associated with obesity and type 2 diabetes. Because the prevalence of obesity is increasing worldwide, the number of patients with NAFLD is also increasing. The spectrum of NAFLD ranges from simple steatosis to nonalcoholic steatohepatitis that manifests as fatty liver with hepatic injury, inflammation, and fibrosis, which may further progress to cirrhosis and even hepatocellular carcinoma. NAFLD begins with the aberrant accumulation of triglycerides in the liver caused by an imbalance between lipid input and output. An increase in free fatty acid (FFA) uptake from the systemic circulation because of increased lipolysis in adipose tissue and from the diet leads to the accumulation of hepatic triglycerides, contributing to NAFLD development. Despite considerable efforts for better understanding obesity-related NAFLD, the precise mechanisms underlying NAFLD are not fully understood. More importantly, there is no approved treatment for NAFLD.

The term autophagy (from the Greek self-eating) was coined by de Duve and Wattiaux^1^ more than 50 years ago to define a process of vacuolization for the transport of intracellular material to the lysosomes to dispose of intracellular waste. The importance of autophagy was recognized by the award of 2 Nobel Prizes for Physiology or Medicine, and the knowledge and number of autophagy-related publications has increased explosively in recent decades. Importantly, autophagy is a mechanism of clearing accumulated intracellular lipids, and its suppression in chronic liver disease is associated with lipid deposition in the development of NAFLD. A previous report by Takehara’s research group showed that Rubicon, a negative regulator of autophagy, is upregulated in NAFLD and inhibits autophagy activity, thereby exacerbating NAFLD development.^2^ In addition, given the increasing significance of interorgan crosstalk for NAFLD pathogenesis, it was tempting to speculate that the release of FFA by adipose tissue autophagy could contribute to the pathogenesis of NAFLD.

In the current issue of *Cellular and Molecular Gastroenterology and Hepatology*, Sakane et al^3^ provide a novel mechanism of adipose tissue autophagy-mediated NAFLD progression. The authors first show that changes in adipose tissue autophagy following high-fat diet feeding are associated with the pathogenesis of NAFLD. High-fat diet feeding caused weight gain and autophagy in epididymal (representing visceral fat) and inguinal white adipose tissue (representing subcutaneous fat), which correlated with decreased Rubicon expression. This observation was also supported by an in vitro experiment showing that in adipocytes, palmitic acid treatment enhanced autophagy and lipolysis. Enhanced autophagy-mediated lipolysis leads to decreased lipid accumulation in adipocytes and increased FFA levels in the circulation, promoting the flux of FFA into hepatocytes, resulting in the increased fat accumulation in the liver.

Sakane et al^3^ further investigated the role of adipose tissue autophagy on the NAFLD pathogenesis using adipocyte-specific Atg7-null (Adipoq-Atg7 null) mice. Atg7 plays a central role in autophagosome formation, and its deficiency causes a decrease in autophagy activity, mediating intracellular fat accumulation.^4^^,^^5^ In the high-fat diet feeding condition, Adipoq-Atg7 null mice showed similar body weight to wild-type control animals but had decreased serum FFA levels and inguinal white adipose tissue hypertrophy. However, the weight of epididymal white adipose tissue was reduced with the increases in cell apoptosis, oxidative stress, and JNK activity. Furthermore, Adipoq-Atg7 null mice showed reduced hepatic fat accumulation, inflammation, and fibrosis compared with wild-type control animals. These findings demonstrated that suppression of autophagy in adipose tissue results in a reduction of lipolysis and delivery of fatty acids to the liver, alleviating NAFLD/nonalcoholic steatohepatitis development.

Some questions remain. The present study did not investigate how autophagy and Rubicon expression are differentially regulated between liver and adipose tissues in NAFLD. In advanced NAFLD, autophagy is impaired, and Rubicon is increased in the liver. In contrast, in adipose tissue, high-fat diet feeding causes increased autophagy along with decreased Rubicon expression. Additional studies are required to clarify the distinct regulatory mechanisms of autophagy and Rubicon expression in adipose tissue and liver. Nevertheless, this study demonstrated a potential role for crosstalk between adipose tissue autophagy and liver pathogenesis in the development of NAFLD/nonalcoholic steatohepatitis. Furthermore, future work in this area may lead to a novel treatment strategy targeting adipose tissue autophagy to combat NAFLD.
